# Editorial: CNS tumor metabolism: targets, markers, and challenges

**DOI:** 10.3389/fncel.2024.1401687

**Published:** 2024-03-27

**Authors:** Beatriz I. Fernandez-Gil, Mioara Larion

**Affiliations:** ^1^Department of Neurological Surgery, Mayo Clinic, Jacksonville, FL, United States; ^2^Neuro-Oncology Branch, Center for Cancer Research, National Cancer Institute (NIH), Bethesda, MD, United States

**Keywords:** central nervous system (CNS), cancer metabolism, GBM, lower-grade glioma (LGG), Deuterium Metabolic Imaging (DMI), Magnetic Resonance Spectroscopy (MRS), epilepsy, subarachnoid hemorrhage

Central nervous system (CNS) cancer ranks as the 10th leading cause of death globally, prompting a critical shift toward cutting-edge techniques and innovative insights into classical approaches. Traditionally, the study of tumor metabolism was reduced to the study of Warburg effect and constrained by technological limitations. However, recent advancements in mass spectrometry have revolutionized our understanding of this field, providing a promising avenue to address this urgent health concern. This journal Research Topic explores diverse metabolic alterations in CNS cancers, ranging from symptomatology effectors to diagnostic predictors and tools. It considers imaging applications, target discovery, and biomarkers, and also delves into new pathways and the metabolic implications on tumor comorbidities.

Beyond classical approaches such as aerobic glycolysis, this Research Topic explores on alternative metabolic pathways in the context of brain tumors. The study by Li et al. focuses on the significant role of tryptophan metabolism in the progression of lower-grade glioma (LGG). They develop a risk model and molecular subtypes based on tryptophan metabolism-related genes, accurately predicting the prognosis of LGG patients. This study highlights the potential of tumoral metabolic assessment in guiding personalized treatment strategies and predicting patient outcomes.

Diagnostic and therapeutic approaches for CNS metabolism are also addressed in this Research Topic. Ip et al. employ Deuterium Metabolic Imaging (DMI) to map exogenous choline uptake in rat glioblastoma (GBM), revealing the potential of DMI-based metabolic maps for characterizing brain tumors. Their work takes advantage of the high uptake and metabolism of exogenous choline in rat glioma cells compared to normal brain, leading to a high tumor-to-brain image contrast on DMI-based metabolic maps. The study demonstrates the potential of using deuterated choline combined with DMI to metabolically characterize brain tumors, which could have a crucial role in understanding the metabolic intricacies of GBM. Importantly, DMI holds potential for personalized medicine by characterizing individual tumor metabolic profiles, enabling the development of targeted treatment strategies based on specific metabolic vulnerabilities.

Additionally, Kamson et al. review the role of lactate as a non-invasive biomarker of dichloroacetate (DCA) therapy. DCA works to reduce lactate production in both cancer and non-cancer central nervous system disorders by stimulating pyruvate oxidation, and subsequent inhibition of glutamine oxidation. This cascade leads to decreased pyruvate availability for transamination, resulting in glutamate accumulation and inhibition of glutaminase activity. This mechanism ultimately leads to a reduction in lactate levels in the tissues, as observed in various studies involving cancer cell lines and animal models. The importance of this reduction lies in the association of lactic acidosis with poorer survival and treatment resistance, particularly in malignancies like GBM. In this context, Magnetic Resonance Spectroscopy (MRS) emerges as a non-invasive and radiation-free technique to monitor metabolic changes in response to DCA, offering insights into drug delivery, tissue response, and therapeutic efficacy in patients with neurologic and oncologic disorders. Techniques such as MRS play a pivotal role in tracking metabolic changes, yielding valuable information on disease severity, treatment response, and prognosis. Lactate's role as a biomarker extends beyond tracking disease progression, also encompassing the assessment of treatment efficacy, prediction of clinical outcomes, and the customization of therapeutic approaches based on individual metabolic profiles. This integrated approach exemplifies the fusion of imaging, prognosis, and therapeutic applications in metabolic studies.

Abnormal metabolic characteristics of tumors can potentially impact the CNS in different ways, increasing the risk of comorbidities. McAfee et al.'s review delves into the pro-epileptic pathological changes in tumor cells, emphasizing metabolic differences between IDH1 mutant and wildtype glioma cells that lead to seizure development. IDH1 mutant cells show reduced glucose consumption, downregulated glycolysis, and dependence on intracellular glutamate breakdown, relying on glutamine as a primary carbon source. These cells overproduce D-2-hydroxyglutarate (D2HG), activating glutamate receptors and fostering hyperexcitability. Conversely, IDH1 wildtype cells upregulate glycolysis, releasing excess glutamate and lactate, contributing to neighboring neuron hyperexcitability. Both cell types induce metabolic shifts, promoting epileptogenesis through unique mechanisms, such as NMDA receptor activation and mTOR pathway engagement in IDH1 mutant cells, and glutamate release, lactate accumulation, and relative peritumoral hypoxia in IDH1 wildtype cells. Understanding these metabolic distinctions is crucial for identifying biomarkers for glioma classification and prognosis, developing targeted therapeutic approaches, and improved surgical planning using non-invasive imaging techniques in glioma patients experiencing seizures.

Finally, the impact of tumors and cancer treatments on cardiovascular health is explored by Chen et al., who investigate cancer-related subarachnoid hemorrhage (SAH) in Chinese and Brazilian populations. Recognizing SAH promptly in cancer patients is emphasized across this multicenter retrospective study, given the common clinical features and poor outcomes. Independent risk factors for SAH onset in cancer patients were identified, improving prediction and management. The study also highlights the association between coagulation dysfunction and SAH in cancer, with metabolic markers like prealbumin and gamma-glutamyl transferase influencing survival. In summary, the study underscores the crucial role of considering metabolic factors in the diagnosis, prognosis, and treatment of cancer-related SAH, with monitoring and addressing metabolic dysfunctions playing a significant role in comprehensive patient care.

Collectively, these studies contribute to a deeper understanding of the metabolic aspects of brain tumors, offering potential prognostic markers and novel avenues for diagnosis and treatment of CNS disorders. The integration of imaging, prognosis, and therapeutic applications exemplifies the evolving field of metabolic studies in CNS cancers, providing a foundation for advancing patient care.

## Author contributions

BF-G: Conceptualization, Writing – original draft, Writing – review & editing. ML: Writing – review & editing.

